# Enhanced Susceptibility of Ogg1 Mutant Mice to Multiorgan Carcinogenesis

**DOI:** 10.3390/ijms18081801

**Published:** 2017-08-18

**Authors:** Anna Kakehashi, Naomi Ishii, Takahiro Okuno, Masaki Fujioka, Min Gi, Hideki Wanibuchi

**Affiliations:** Department of Molecular Pathology, Osaka City University Graduate School of Medicine, Asahi-machi 1-4-3, Abeno-ku, Osaka 545-8585, Japan; m1159070@med.osaka-cu.ac.jp (N.I.); m2026860@med.osaka-cu.ac.jp (T.O.); m2066048@med.osaka-cu.ac.jp (M.F.); mwei@med.osaka-cu.ac.jp (M.G.); wani@med.osaka-cu.ac.jp (H.W.)

**Keywords:** oxoguanine glycosylase 1 (*Ogg1*), 8-hydroxy-2′-deoxyguanosine, DNA repair, multiorgan carcinogenesis bioassay

## Abstract

The role of deficiency of oxoguanine glycosylase 1 (*Ogg1*) *Mmh* homolog, a repair enzyme of the 8-hydroxy-2’-deoxyguanosine (8-OHdG) residue in DNA, was investigated using the multiorgan carcinogenesis bioassay in mice. A total of 80 male and female six-week-old mice of C57BL/6J background carrying a mutant *Mmh* allele of the *Mmh/Ogg1* gene (*Ogg1*^−/−^) and wild type (*Ogg1*^+/+^) mice were administered *N*-diethylnitrosamine (DEN), *N*-methyl-*N*-nitrosourea (MNU), *N*-butyl-*N*-(4-hydroxybutyl) nitrosamine (BBN), *N*-bis (2-hydroxypropyl) nitrosamine (DHPN) and 1,2-dimethylhydrazine dihydrochloride (DMH) (DMBDD) to induce carcinogenesis in multiple organs, and observed up to 34 weeks. Significant increase of lung adenocarcinomas incidence was observed in DMBDD-treated *Ogg1*^−/−^ male mice, but not in DMBDD-administered *Ogg1*^+/+^ animals. Furthermore, incidences of lung adenomas were significantly elevated in both *Ogg1*^−/−^ males and females as compared with respective *Ogg1*^−/−^ control and DMBDD-treated *Ogg1*^+/+^ groups. Incidence of total liver tumors (hepatocellular adenomas, hemangiomas and hemangiosarcomas) was significantly higher in the DMBDD-administered *Ogg1*^−/−^ males and females. In addition, in DMBDD-treated male *Ogg1*^−/−^ mice, incidences of colon adenomas and total colon tumors showed a trend and a significant increase, respectively, along with significant rise in incidence of simple hyperplasia of the urinary bladder, and a trend to increase for renal tubules hyperplasia in the kidney. Furthermore, incidence of squamous cell hyperplasia in the forestomach of DMBDD-treated *Ogg1*^−/−^ male mice was significantly higher than that of *Ogg1*^+/+^ males. Incidence of small intestine adenomas in DMBDD *Ogg1*^−/−^ groups showed a trend for increase, as compared to the wild type mice. The current results demonstrated increased susceptibility of *Ogg1* mutant mice to the multiorgan carcinogenesis induced by DMBDD. The present bioassay could become a useful tool to examine the influence of various targets on mouse carcinogenesis.

## 1. Introduction

DNA damage and disruption of DNA repair are considered key factors in the susceptibility of mammals to endogenous and exogenous carcinogens, as well as processes of aging and cancer development [1]. The oxidative DNA damage includes a variety of oxidative lesions in DNA and the main attack site of reactive oxygen species (ROS) is at the 8 position of guanine, producing strongly mutagenic base 8-hydroxy-2′-deoxyguanosine (8-OHdG) [2]. 8-OHdG is used as an oxidative DNA damage marker which mispairs with adenine (A) residues, thus resulting in increase of spontaneous G:C to T:A transversion mutations [3].

Three DNA repair enzymes from various bacteria and *Saccharomyces cerevisiae*, namely, the MutM (Fpg), MutY and MutT DNA glycosylase homologs are known to prevent spontaneous mutagenesis induced by 8-OHdG [4]. In mammalian cells, the MutM homolog (MMH; the glycosylase/apurinic, apyrimidinic (AP) lyase), MutY and MutT homolog enzymes have also been identified [5,6,7]. In both mammalian and yeast cells, cloned human and mouse cDNAs encode distinct nuclear and mitochondrial forms of the DNA glycosylase, the product of the *Ogg1* gene, which is generated by alternative RNA splicing [8,9,10]. MutY and MutM homologs prevent G:C to T:A transversions in DNA, while MutT protein hydrolyzes 8-oxo-dGTP to 8-oxo-dGMT and pyrophosphate, thus avoiding the occurrence of A:T to C:G transversion mutations during DNA replication [11,12]. Analysis of the mutation spectrum revealed that the frequency of G:C to T:A transversions increased five-fold in *Ogg1* mutant mice compared with wild-type animals [8].

*Mmh/Ogg1* homozygous mutant (*Ogg1*^−/−^) mice used in our studies have physically normal appearance but exhibit three- and seven-fold increased accumulation of 8-OHdG adduct at 9 and 14 weeks of age, respectively, in comparison with or heterozygous or wild-type animals [13]. We have previously demonstrated that treatment of *Ogg1*^−/−^ mice with dimethylarsinic acid (DMA) and phenobarbital (PB) for 78 weeks resulted in enhancement of lung and liver carcinogenesis, respectively [14,15]. The tremendous increase of 8-OHdG levels with consequent G:C to T:A transversions and deletions in the kidney DNA of *Ogg1*^−/−^ mice were reported following administration of potassium bromate (KBrO_3_) [8]. Furthermore, a significant increase of mutation frequency in *Ogg1*^−/−^ mice livers was observed during liver regeneration after partial hepatechtomy following KBrO_3_ treatment [16]. In addition, Sakumi et al. and Xie et al. demonstrated spontaneous development of lung, ovary tumors and lymphomas in *Myh* and *Ogg1* knockout mice [5,17]. However, it is still unknown how the ablation of these enzymes affects the tumorigenicity of various chemical carcinogens.

Previously, several in vivo bioassay systems for carcinogenicity detection of test compounds have been developed. However, these bioassays usually predict carcinogenicity of test chemicals only in single organs with known strategies of carcinogenesis initiation. To develop the experimental approach for the determination of carcinogenicity in numerous target organs, multiorgan wide-spectrum initiation bioassay (namely, the multiorgan carcinogenicity bioassay: DMBDD model) has been established [18,19,20,21]. This bioassay was applied in rats and included treatment with five genotoxic carcinogens, *N*-diethylnitrosamine (DEN), *N*-methyl-*N*-nitrosourea (MNU), *N*-butyl-*N*-(4-hydroxybutyl) nitrosamine (BBN), *N*-bis (2-hydroxypropyl) nitrosamine (DHPN) and 1,2-dimethylhydrazine dihydrochloride (DMH) (DMBDD), as initiators of liver, lungs, kidneys, urinary bladder, stomach, small intestine, colon and thyroid gland carcinogenesis [19,22,23]. It has been demonstrated that DMBDD-induced organ-specific DNA damage could be attributed to free radicals, methylation, and accumulation of non-repaired DNA damage [23]. In rats, DEN is usually used as initiator of liver carcinogenesis, BBN as initiator of bladder carcinogenesis, DMH as initiator of intestine carcinogenesis, and MNU as initiator of stomach, bladder and liver carcinogenesis [24]. DHPN is a wide-spectrum carcinogen in rats, which induces lung, thyroid, kidney, bladder and liver cancers [23,25]. In previous studies, DMBDD treatment has been proposed to inactivate the tumor suppressor p53 in the bladder tumors of Zucker diabetic rats [26]. However, to our knowledge, the DMBDD model was never applied in mice and there is no information how the DMBDD treatment influences oncogenes and tumor suppressor genes.

The aim of the present study was to investigate the differences in susceptibility of *Ogg1* mutant and wild type mice of C57BL/6J background to the treatment with five types of genotoxic carcinogens (DEN, MNU, BBN, DHPN and DMH: DMBDD) by applying the multiorgan carcinogenesis bioassay in mice.

## 2. Results

### 2.1. General Observations

All control *Ogg1*^−/−^ male or female mice were alive at the end of the study. They were healthy and long-lived as compared to the control *Ogg1*^+/+^ mice. Three DMBDD-treated *Ogg1* knockout male and three female mice were found moribund at Weeks 11, 12, and 17, and 10, 20 and 25, respectively. The causes of death of *Ogg1*^−/−^ male mice were malignant lymphomas/leukemia, lung adenocarcinoma and fibrosarcoma, while *Ogg1*^−/−^ female mice died due to the development of lymphoma/leukemia. Four DMBDD-administered *Ogg1*^+/+^ male and one female mice died at Weeks 16, 29, 33, 35 and 32, respectively. The main causes of death in male and female wild type mice were malignant lymphoma/leukemia, T cell lymphoma and bladder transitional cell carcinoma (TCC). One non-treated control *Ogg1*^+/+^ male mouse was found moribund at Week 27 due to a urinary tract infection.

**Figure 1:**
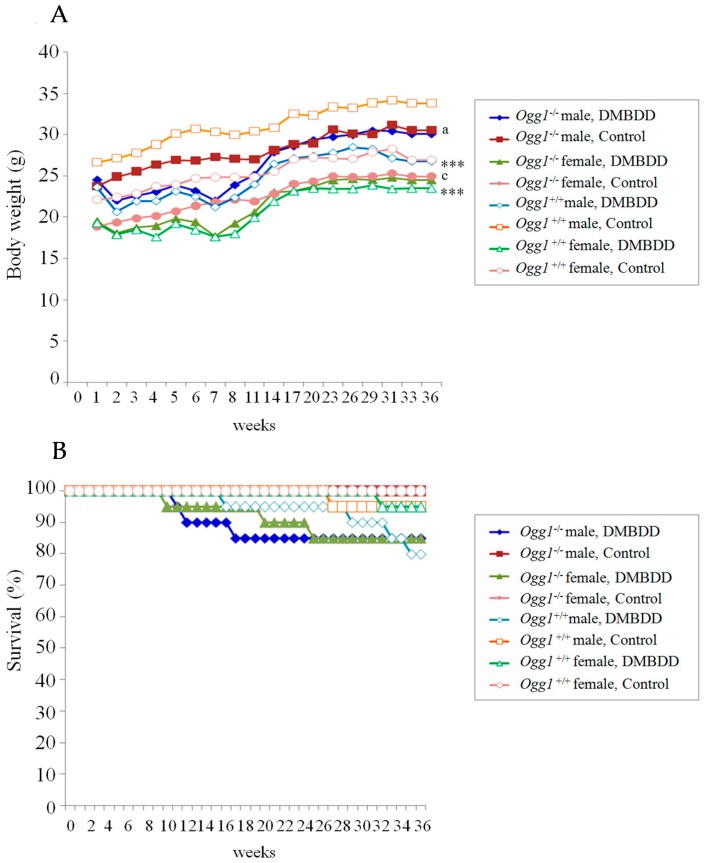
Body weight (**A**); and survival (**B**) curves for DMBDD-treated and control *Ogg1*^−/−^ and *Ogg1*^+/+^ male and female mice. *** *p* < 0.001 significantly different vs. respective control group of the same genotype; ^a^ *p* < 0.05 and ^c^ *p* < 0.001 significantly different vs. the respective *Ogg1*^+/+^ control groups.

As lung, liver and colon tumors were observed in the DMBDD-treated *Ogg1*^−/−^ mice that were found moribund during the study, effective number of animals used for the histopathological analysis included all mice. Body weight and survival curves, final body weight and absolute and relative organ weights of mice are shown in Table 1 and Figure 1. Body weights of control *Ogg1* mutant male and female mice were significantly lower than those of wild type mice all through the experiment. DMBDD treatment induced significant decrease of body weight of both *Ogg1* mutant and wild type mice, However, at Experimental Week 14, mean body weight of *Ogg1*^−/−^ mice became equal to that of the corresponding control animals of the same genotype, while the body weight of the DMBDD-administered *Ogg1*^+/+^ mice continued to be significantly lower compared to the control *Ogg1*^+/+^ until the end of the study (Figure 1A). Therefore, at Week 34, final body weight of the DMBDD-treated *Ogg1*^+/+^ but not *Ogg1*^−/−^ mice were significantly decreased as compared with the control mice of the same genotype.

DMBDD administration inhibited food intake of the *Ogg1*^+/+^, but not the *Ogg1*^−/−^ mice compared with the control mice of the same genotype (Figure S1A). Water intakes were similarly decreased in all DMBDD-treated *Ogg1*^−/−^ and *Ogg1*^+/+^ animals (Figure S1B). Thus, the body weight of the DMBDD-administered *Ogg1*^+/+^ mice appeared to be significantly lower than the control *Ogg1*^+/+^ due to the inhibited food intake. The absolute and relative liver, kidneys and spleen weights of the control *Ogg1*^−/−^ male and female mice were significantly lower than those of the control *Ogg1*^+/+^ mice (Table 1). DMBDD treatment induced significant increases of relative liver, kidneys and spleen weights of *Ogg1* mutant but not wild type both male and female mice in comparison with corresponding *Ogg1^−/−^* controls. The absolute and relative weights of the lungs were significantly increased in both *Ogg1^−/−^* and *Ogg1^+/+^* male and female mice (Table 1).

### 2.2. Survival Curves

Survival curves for the DMBDD-administered and control *Ogg1^−/−^* and *Ogg1^+/+^* mice are presented in Figure 1B. In the present model, there were no significant differences in survival between the control *Ogg1* homozygous mutant and wild type mice. Trends for decrease in survival were observed in both *Ogg1^−/−^* and *Ogg1*^+/+^ DMBDD-treated animals. However, in DMBDD-treated *Ogg1*^−/−^ male and female mice earlier decrease in survival (males: Week 11; females: Week 10), respectively, as compared to the wild type mice (males: Week 16; females: Week 32) was found (Figure 1B). Importantly, the earlier development of tumors in DMBDD-administered *Ogg1*^−/−^ males and females, mostly malignant lymphomas/leukemias, lung adenocarcinoma and subcutaneous tumors (fibrosarcomas) was the reason for their earlier mortality.

### 2.3. Results of Histopathological Examination

**Figure 2:**
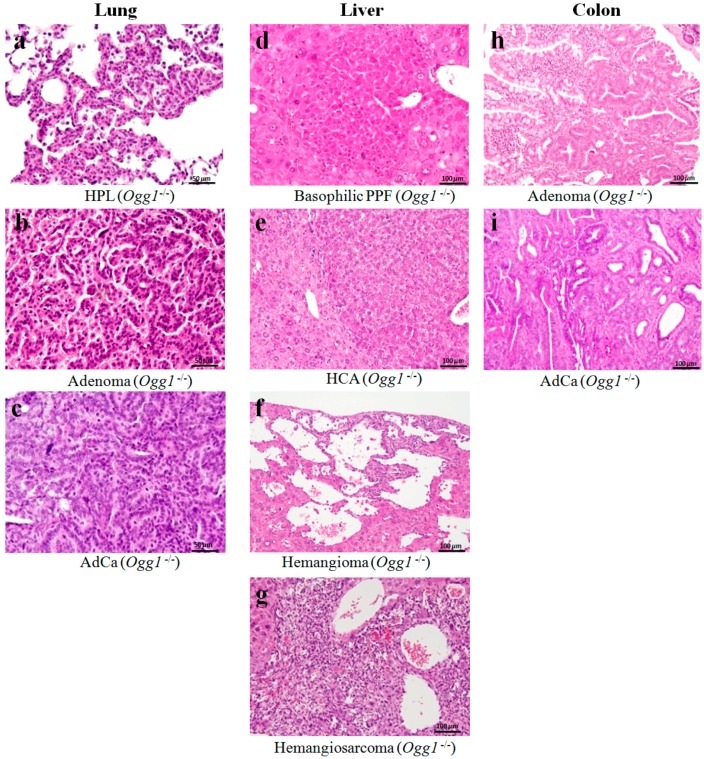
Representative histopathological pictures (H&E staining) of: lung hyperplasia (**a**); adenoma (**b**); adenocarcinoma (**c**); liver PPF (basophilic foci) (**d**); HCA (**e**); hemangioma (**f**); hemangiosarcoma (**g**); colon adenoma (**h**); and adenocarcinoma (**i**) developed in DMBDD-treated *Ogg1*^−/−^ mice. HPL, hyperplasia; HCA, hepatocellular adenoma; AdCa, adenocarcinoma; PPFs, putative preneoplastic foci.

Table 2 summarizes the data on the incidence of preneoplastic, neoplastic and some non-neoplastic lesions and general distribution of tumors induced by DMBDD administration in *Ogg1* knockout and wild type mice. Representative pictures of neoplastic lesions observed in the lungs, livers and colons of mice are presented in Figure 2. Neoplastic nodules induced in the DMBDD-treated group of *Ogg1*^−/−^ and *Ogg1*^+/+^ mice were mainly lung, liver, colon, small intestine, urinary bladder tumors, malignant lymphomas/leukemias and subctaneous tumors (fibrosarcomas). In male *Ogg1*^+/+^ mice higher number of tumors were induced by the DMBDD treatment as compared to the *Ogg1*^+/+^ females likely due to the lower susceptibility to genotoxic carcinogens in females.

Macroscopically, no tumors were found in the non-treated control *Ogg1*^+/+^ mice, however, spontaneous development of lung nodules (hyperplasia and adenoma) was detected in the control *Ogg1*^−/−^ animals. Furthermore, DMBDD-treated *Ogg1*^−/−^ mice were more susceptible to the induction of different tumors as compared to *Ogg1*^−/−^ control and wild type mice. DMBDD treatment induced elevation of total tumor incidence and number of tumor bearing mice in mutant, predominantly *Ogg1*^−/−^ female animals (males, 100%, 5.9 ± 3.5/mouse; females, 100%, 4.6 ± 2.1/mouse, *p* < 0.0001), as compared to the wild type mice (males, 85%, 3.5 ± 2.8/mouse; females, 40%, 0.7 ± 0.9/mouse). All DMBDD-treated male and female *Ogg1*^−/−^ mice developed many nodules in the lungs, while incidences and multiplicities of lung nodules in DMBDD-treated *Ogg1*^+/+^ was lower as compared to the *Ogg1*^−/−^ DMBDD-administered animals. Furthermore, the incidence of liver lesions in *Ogg1*^−/−^ mice, as well as their multiplicity was also increased after carcinogens treatment as compared to the corresponding controls. Moreover, in male, but not female DMBDD-treated *Ogg1*^−/−^ mice, incidences of colon tumors and fibrosarcomas, were elevated.

Histopathological examination demonstrated significant elevation of incidences and multiplicities of lung adenoma and total lung tumors in DMBDD-treated *Ogg1*^−/−^ male and female mice lungs (total tumors: males, 100%, 4.1 ± 2.7/mouse; females, 100%, 4.1 ± 2.2/mouse; adenoma: males, 100%, 4.0 ± 2.3/mouse; females, 95%, 2.7 ± 2.8/mouse) as compared to both respective *Ogg1*^−/−^ controls (total tumors: males, 5%, 0.1 ± 0.2/mouse; females, 0%, 0/mouse; adenoma: males, 5%, 0.1 ± 0.2/mouse; females, 0%, 0/mouse ) and DMBDD-treated *Ogg1*^+/+^ mice (total tumors: males, 75%, 3.0 ± 2.7/mouse; females, 60%, 1.4 ± 2.2/mouse; adenoma: males, 70%, 2.7 ± 2.8/mouse; females, 60%, 1.4 ± 2.0/mouse) (Table 2). Interestingly, significant increase of lung adenocarcinoma incidence and multiplicity was found in DMBDD-administered *Ogg1*^−/−^ male mice (35%, 0.5 ± 0.7/mouse, *p* < 0.01), but not in the wild type males (10%, 0.1 ± 0.3/mouse) as compared to corresponding controls of the same genotype. Furthermore, incidences and multiplicities of lung adenocarcinoma were higher in female *Ogg1*^−/−^ mice of DMBDD group as compared to the DMBDD-treated wild type counterparts. In addition, increases of lung hyperplasia incidences due to the DMBDD application were observed in both *Ogg1* homozygous mutant and wild type mice.

In the liver of *Ogg1* knockout and wild type mice, DMBDD treatment caused development of putative preneoplastic foci of mostly basophilic phenotype, hepatocellular adenomas (HCAs), hemangiomas and hemangiosarcomas. Importantly, hemangiosarcomas were detected only in the *Ogg1*^−/−^ mice (males, 15%, 0.6 ± 1.4/mouse; females, 5%, 0.1 ± 0.2/mouse). No hepatocellular carcinomas were apparent in DMBDD-treated *Ogg1* knockout and wild type animals. Increases of HCA incidences (males, 20%; females, 20%; *p* = 0.05) and multiplicities (males: 0.3 ± 0.6/mouse, *p* = 0.05; females, 0.2 ± 0.4) were detected in livers of the DMBDD-initiated *Ogg1*^−/−^ mice as compared to the *Ogg1*^−/−^ controls (males, 0%, 0.1 ± 0.2/mouse; females, 0%, 0/mouse) and DMBDD-treated *Ogg1*^+/+^ groups (males, 5%, 0.1 ± 0.2/mouse; females, 0%, 0/mouse). Significant elevations of total liver tumor incidences were observed in *Ogg1*^−/−^ males (25%, *p* < 0.05) and females (25%, *p* < 0.05), but not *Ogg1*^+/+^ mice as compared to the corresponding controls of the same genotype (0%). Furthermore, a trend for increase and a significant elevation of total liver tumors multiplicity were observed in DMBDD-treated *Ogg1*^−/−^ males (0.9 ± 2.0/mouse, *p* = 0.05) and females (0.3 ± 0.6/mouse, *p* < 0.05), in respect of control *Ogg1*^−/−^ group. In addition, DMBDD administration caused elevation of bile duct proliferation in the liver of *Ogg1*^−/−^ mice as compared to the *Ogg1*^−/−^ and *Ogg1*^+/+^ counterparts. Significant increases of biliary cysts formation in the DMBDD groups were observed in the liver of both *Ogg1*^−/−^ and *Ogg1*^+/+^ animals in respect of corresponding controls (Table 2).

In kidneys, significant increase of renal tubular degeneration (80%, *p* < 0.05) and a trend for increase of tubular renal cell HPL was found in the DMBDD-treated *Ogg1*^−/−^ male mice as compared to corresponding controls of the knockout and wild type genotypes (Table 2). Only one *Ogg1*^+/+^ DMBDD-treated mouse developed renal adenoma.

In the urinary bladder, significant increase of simple hyperplasia (25%, *p* < 0.05) and a trend for increase of papillary and nodular (PN) hyperplasia (20%) incidences as compared to the corresponding controls of the same genotype was detected (Table 2).

The incidence of total colon tumors (25%, *p* < 0.05) was significantly increased in the DMBDD-treated *Ogg1*^−/−^ male mice but not in the DMBDD-administered wild type males (10%). Development of adenocarcinoma (5%) was found in one male *Ogg1*^−/−^ mouse of the DMBDD group. Furthermore, incidences of small intestine total tumors showed a trend for increase in the DMBDD-administered *Ogg1*^−/−^ male mice (20%) as compared to the *Ogg1*^−/−^ control group and DMBDD-treated *Ogg1*^+/+^ animals (5%). In females, incidences and multiplicities of colon tumors induced by DMBDD were comparable with that of observed in wild type mice, pointing out the sex differences in susceptibility to colonic tumorigenesis.

In the forestomach, the DMBDD treatment resulted in significant elevation of the squamous cell HPL incidence in *Ogg1*^−/−^ (male, 70%, *p* < 0.0001; female, 55%, *p <* 0.001) and *Ogg1*^+/+^ (male, 35%, *p* < 0.01; female, 40%, *p <* 0.01) mice as compared to the corresponding controls of the same genotype. Furthermore, in male mice, it was significantly increased in comparison to wild type DMBDD-treated males (*p* < 0.05).

Trends for increase of malignant lymphomas/leukemias were observed in *Ogg1* homozygous mutant males (15%) and females (20%) treated with DMBDD, as compared to wild type mice (Table 2). One *Ogg1*^+/+^ female mouse in DMBDD group developed T cell lymphoma (5%).

### 2.4. Blood Biochemistry

The results of the blood biochemistry analysis are shown in Table 3. Aspartate aminotransferase (AST) and alanine aminotransferase (ALT) levels in the blood of both *Ogg1* null and wild type mice showed strong trends for increase, or were significantly elevated by the DMBDD treatment. Furthermore, this induction was higher in *Ogg1*^−/−^ animals. Serum sodium (Na) levels were elevated by the DMBDD administration in both *Ogg1* mutant and wild type mice. Moreover, creatinine level was higher in the blood of DMBDD-treated *Ogg1*^−/−^ mice as compared to the respective *Ogg1*^−/−^ control groups. Alkaline phosphatase (ALP), T-cholesterol and chloride (Cl) levels were lowered in the wild type DMBDD-treated animals, but not altered in DMBDD *Ogg1*^−/−^ mice. Serum calcium (Ca) level was significantly decreased in the DMBDD-treated *Ogg1* knockout male mice as compared to the wild type males administered DMBDD. In addition, inorganic phosphorus (IP) levels showed a trend for increase or the significant elevation in the blood of DMBDD-treated and control *Ogg1*^−/−^ male and female mice, respectively, as compared to the wild type groups receiving the same treatment.

In the blood serum of *Ogg1* mutant and wild type mice, levels of total protein showed a trend (*Ogg1*^−/−^ males, females and *Ogg1*^+/+^ males) and a significant decrease (*Ogg1*^+/+^ females) as compared to the non-treated respective control groups. Furthermore, albumin levels were lower in DMBDD-treated *Ogg1*^−/−^ and *Ogg1*^+/+^ groups, with significant differences observed for *Ogg1*^−/−^ and *Ogg1*^+/+^ DMBDD-administered males. Albumin/globulin (A/G) ratio was significantly lower in the *Ogg1*^+/+^ male DMBDD group.

## 3. Discussion

The present study revealed that *Ogg1* mutant mice are more susceptible to the induction of tumors due to the treatment with DMBDD, than wild type C57Bl/6J mice. In the DMBDD-treated *Ogg1*^−/−^ mice, main causes of death besides malignant lymphoma/leukemia were lung adenocarcinoma and skin/subcutis fibrosarcoma, while *Ogg1*^+/+^ animals died from malignant lymphoma/leukemia and urinary bladder carcinoma. Furthermore, the earlier mortality of DMBDD-administered *Ogg1*^−/−^ mice appeared to be due to the earlier tumor development. Importantly, DMBDD caused significant increases of incidences and multiplicities of lung adenocarcinoma in *Ogg1*^−/−^ males, liver tumors in *Ogg1*^−/−^ males and females and colon tumors in *Ogg1*^−/−^ male mice as compared to the *Ogg1*^−/−^ controls. In the kidneys, urinary bladder, stomach, small intestine and subcutis of *Ogg1* mutant mice, increases of carcinogenicity as compared to the DMBDD-treated wild type animals were obvious.

Lungs of *Ogg1* null mice were strongly affected by DMBDD initiation, which could be concluded from significant increases of lung adenocarcinoma incidence in DMBDD-treated *Ogg1*^−/−^ male mice and incidences and multiplicities of adenomas and total lung tumors in *Ogg1*^−/−^ males and females. As lungs are strongly exposed to molecular oxygen, it is likely the most carcinogenicity sensitive organ in *Ogg1* knockouts. Furthermore, in the lung of non-treated *Ogg1*^−/−^ animals, spontaneously developed tumors were observed, possibly due to the accumulation of non-repaired oxidative DNA base modifications even in the absence of initiation. Several authors have reported an increase of spontaneous lung tumors in MutM, MutY and MutT-deficient mice [5,14,15,27]. Previously, lung tumors were also shown to be significantly induced in *Ogg1*^−/−^ mice by the DMA treatment [14]. Furthermore, significant enhancement of spontaneous lung tumorigenesis was observed when the *Ogg1* mutation was combined with a MutY homolog (MUTYH) or MSH2-deficient condition, and the G:C to T:A transversions in the *K-ras* gene were detected in the lung tumors [17]. In our previous study, genes related to cancer, cellular growth, proliferation and cell cycle (e.g., polymerase (DNA-directed), delta 4 (Pold4), cyclin C and mitogen activated protein kinase 8) and angiogenesis (e.g., matrix metalloproteinases 13, 14, and 17) were found to be up-regulated in non-treated *Ogg1*^−/−^ mice lungs, but those involved in free radical scavenging, lipid metabolism, drug and endocrine system development and function were suppressed comparing to the *Ogg1*^+/+^ case [14]. From the present and previous results, MutM, MutY and MutT homologs responsible for the repair of oxidative DNA modifications are extremely important for suppression of lung tumorigenesis in mammal.

**Figure 3:**
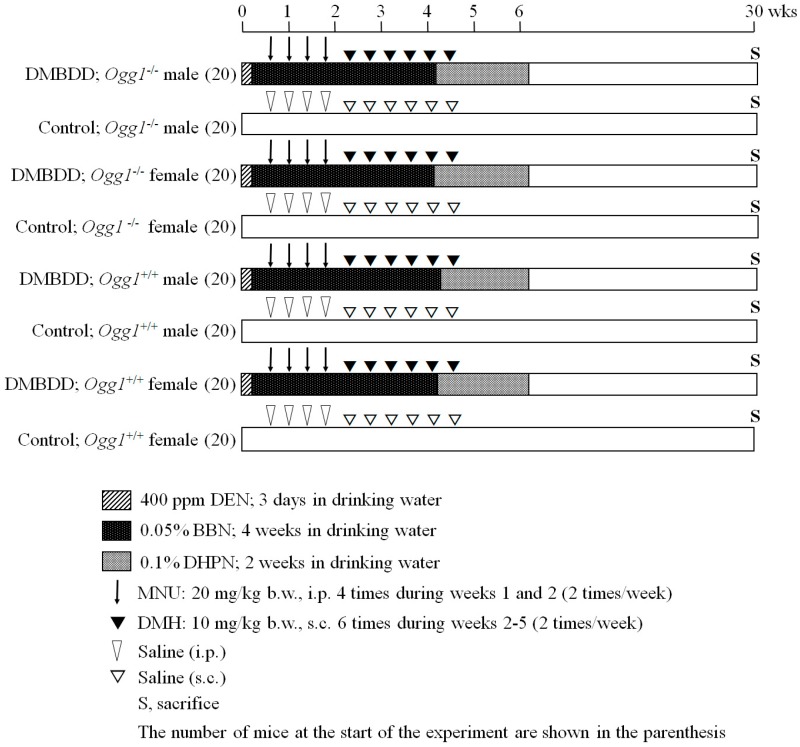
Experimental protocol of medium-term multiorgan carcinogenesis bioassay applied in *Ogg1*^−/−^ and *Ogg1*^+/+^ mice. wks: weeks.

DMBDD treatment induced significant increases of relative liver weights in *Ogg1* homozygous mutant but not wild type mice. Furthermore, higher elevation of AST and ALT serum levels reflecting the pathological processes in the liver supported our histopathological findings in the DMBDD-treated *Ogg1* knockout mice. The mechanism of DMBDD carcinogenicity in the liver of *Ogg1*^−/−^ mice might be accumulation of non-repaired oxidative base modifications in DNA leading to increase of cell proliferation, occurrence of mutations and further elevation of cell proliferation, resulting in promotion and progression of liver carcinogenesis [14]. Interestingly, hemangiosarcomas were detected only in the DMBDD-treated *Ogg1*^−/−^ mice. Hemangiomas and hemangiosarcomas are known to arise as primary vascular neoplasms in the liver and could be initiated in mice by DHPN [28]. They are usually not sharply demarcated from the surrounding parenchyma and the neoplastic cells are generally elongated or spindle-shaped and may form solid areas occupying dilated hepatic sinusoids and are typically locally invasive (Figure 3). Tsutsumi et al. previously demonstrated that incidences and multiplicities of hemangiomas and hemangiosarcomas in the liver were markedly higher in the poly(ADP-ribose) polymerase-1 (Parp-1)-null mice, while Parp-1 is one of the poly(ADP-ribose) polymerase family proteins taking part in genomic stability, DNA repair and cell death triggered by DNA damage [29]. Thus, the relationship between defective DNA repair and development of hemangiosarcomas may exist.

Increase of DNA 8-OHdG levels has been previously reported by the DEN treatment in the livers of rats and mice [3,30]. Furthermore, *mutT*-deficient mice were also reported to be susceptible to liver carcinogenesis [27]. Moreover, the effect of potassium bromate, which has been reported to induce oxidative stress, was investigated in *Ogg1*^−/−^ mouse liver after partial hepatectomy [16], and the results indicated a significant increase of mutation frequency and liver tumorigenicity being consistent with our present and previous data showing the promotion and progression of hepatocarcinogenesis in DMBDD and PB-treated *Ogg1*^−/−^ mice [15]. Arai et al. suggested that high levels of cell proliferation are very important for the fixation of mutations induced by oxidative stress conditions in the liver [16]. Furthermore, in our previous study, it has been detected that cell proliferation and DNA 8-OHdG levels in the liver of *Ogg1*^−/−^ mice treated with PB are much higher than that of wild type mice. Therefore, they are highly susceptible to the carcinogens treatment [15]. Thus, it could be suggested that accumulation of unrepaired 8-OHdG in the livers of DMBDD-treated *Ogg1*^−/−^ animals might cause a significant increase of cellular proliferation, resulting in acceleration of hepatocarcinogenesis. With regard to specific elevation of cell proliferation in DMBDD target organs, elevation of cell proliferation has been previously shown in the lung, liver, colon, urinary bladder, thyroid and kidney by initiation with BBN, DEN, DMH, DHPN and MNU [31].

From our previous results, in contrast to the wild type mice, in the livers of *Ogg1*-deficient animals, Nrf2 phosphorylation, and likely, its transformation to the nuclear did not occur, resulting in increase of oxidative stress and DNA damage of liver cells [15]. The accumulation of reactive oxygen species and non-repaired DNA oxidative base modifications in the *Ogg1*^−/−^ livers, thus, could become the reason of higher susceptibility to liver tumorigenesis. At present, Nrf2 is recognized as important protein involved in regulation of broad transcriptional response preventing DNA, proteins and lipids damage, recognition, repair and removal of macromolecular damage, and tissue renewal after application of toxic substance. Mice that lack the Nrf2 transcription factor were more sensitive to the genotoxic and cytotoxic and effects of foreign chemicals and oxidants than wild-type animals [32]. Multiple studies demonstrated enhanced tumorigenicity in Nrf2-disrupted mice compared to wild-type in models of lung disease and cancer, hepatocarcinogenesis, colon cancer, stomach cancer, bladder cancer, mammary cancer, skin cancer, and inflammation [33]. Furthermore, Nrf2 has been shown to upregulate the activity of multiple DNA repair, including the process of removal of oxidative stress-induced endogenous DNA interstrand cross-links [33,34].

The histological examination revealed a trend and a significant increase of renal tubular hyperplasia and degeneration, respectively, in DMBDD-treated *Ogg1*^−/−^, predominantly male mice. The observation of an increased kidney weights and blood biochemistry data supported the finding concerning serious kidneys dysfunction in *Ogg1* mutant mice administered DMBDD. It has been previously suggested that *Ogg1* plays a major role in renal tumorigenesis [35], thus the observed increase of renal tubular hyperplasia could be related to the insufficient repair of 8-OHdG in kidneys. Furthermore, significantly elevated sodium (Na), creatinine and IP level and lowered calcium (Ca) in the blood serum of DMBDD-initiated Ogg1^−/−^ mice signified about the impaired kidney function or kidney disease.

It has been reported that the incidence of bladder cancer induced by BBN is significantly higher in C57BL/6 mouse strains [36]. In this study, we observed development of simple, preneoplastic nodular (PN) hyperplasia and TCC in both DMBDD-administered *Ogg1* homozygous mutant and wild type mice. However, a trend either significant increase for PN and simple urinary bladder hyperplasia incidences was observed, indicating increased susceptibility to bladder carcinogenesis in DMBDD-treated *Ogg1*^−/−^ male and female mice.

In DMBDD-treated *Ogg1*^−/−^ male mice, significantly enhanced incidence and multiplicity of colon tumors as compared to the *Ogg1*^−/−^ control has been found. However, in females, inductions of colon tumors induced by DMBDD in *Ogg1*^−/−^ and *Ogg1*^+/+^ animals were comparable, pointing out the sex differences in susceptibility to colonic tumorigenesis. Furthermore, in our study, an increase of carcinogenicity in the small intestine of *Ogg1*^−/−^ male mice was also observed. Previously, the MutY homolog (MUTYH)-null mice have been reported to have a higher susceptibility to intestinal adenoma and adenocarcinoma [37]. Thus, both the MutM and MutY deficiency leading to high levels of 8-OHdG in the colonic mucosa could be responsible for the tumorigenesis in the colon and small intestine.

In the study with *mutT* homolog-1 (MTH1)-deficient mice, 18 months after birth, increases of tumorigenicity were also detected in stomachs, as compared with wild type mice [27]. These data support our results on enhancement of forestomach squamous cell HPL in DMBDD- treated *Ogg1*^−/−^ male mice, suggesting that *mutT* and *Ogg1* deficiency may promote carcinogenesis in the forestomach.

*Ogg1*-null mice have been reported to show an increased susceptibility to UVB-induced skin tumorigenesis [38]. They developed more malignant tumors (squamous cell carcinomas and sarcomas) than did wild-type mice. In line with these results, in the present study, we observed increase of fibrosarcoma incidence in DMBDD-treated *Ogg1*^−/−^ male mice. Furthermore, trends for increase of incidences of malignant lymphomas/leukemias induced by the DMBDD treatment in *Ogg1*^−/−^ mice as compared to the *Ogg1*^−/−^ controls and DMBDD-treated *Ogg1*^+/+^ animals were found. One DMBDD-treated female *Ogg1*^+/+^ mouse developed T cell lymphoma, likely due to the MNU treatment, as previously reported in C57Bl/6J mice [39], but in our study no such thymic lymphomas were observed in *Ogg1*^−/−^ mice. It is necessary to mention, that no increased risk of thyroid cancer in DMBDD-treated *Ogg1* knockout mice was found in this study.

It is important to note that mutations in the tumor genome induced by the Ogg1 deficiency could also cause tumors to express large number of mutant tumor specific proteins (neoantigens) which have been recently demonstrated to become one of key elements for efficacy of immuno-checkpoint inhibitors as anticancer therapeutics [40].

In conclusion, this study provides the experimental evidence for a strong relationship between repair of the oxidative base modifications and multiorgan carcinogenesis. The mechanism of DMBDD carcinogenicity in the tissues of *Ogg1*^−/−^ mice could be related to the accumulation of non-repaired oxidative DNA modifications leading to mutations and elevation of cell proliferation what likely resulted in promotion and progression of carcinogenesis. The multiorgan carcinogenesis bioassay is concluded to become an important tool to examine the effects of different factors on carcinogenicity in mice.

## 4. Materials and Methods

### 4.1. Chemicals

DEN, BBN and DMH (purity ≥ 98%) were purchased from Tokyo Chemical Industry Co., Ltd. (Tokyo, Japan). DHPN and MNU were purchased from Nacalai Tesque Inc. (Kyoto, Japan) and Wako Pure Chemicals Industries (Osaka, Japan), respectively. Other chemicals were from Sigma or Wako Pure Chemical Industries (Osaka, Japan).

### 4.2. Animals

*Mmh/Ogg1* homozygous mutant (*Ogg1*^−/−^) generated previously [13] and wild type mice (*Ogg1*^+/+^) of C57Bl/6J background were bred and placed in an environmentally controlled room maintained at a constant temperature of 22 ± 1 °C, relative humidity of 44 ± 5% and 12 h (7:00–19:00) light/dark cycle. During all the experimental period they were given free access to drinking water and food (Oriental CE-2 pellet diet, Oriental Yeast Co., Tokyo, Japan). Mice body weights, food and water consumptions were measured weekly for the first 12 weeks of the study and subsequently once every 4 weeks. The time when the animal should be euthanized was decided due to the specific signs, such as no response to stimuli or the comatose condition, loss of body weight loss and related changes in food and water consumption, hypothermia, heart rate and external physical appearance changes, dyspnea and prostration. The experiments were performed according to the Guidelines of the Public Health Service Policy on the Humane Use and Care of Laboratory Animals and approved by the Institutional Animal Care and Use Committee of Osaka City University Graduate School of Medicine (Approval No.597; 26 November 2015).

### 4.3. Experimental Design

We developed the new protocol for multiorgan carcinogenicity bioassay which could be applied in mice (Figure 3). In the present study, *Ogg1*^−/−^ (80) and *Ogg1*^+/+^ (80) six-week-old male and female mice were randomly divided into 4 groups each comprising of 20 mice. The treatment with five genotoxic carcinogens, including DEN, MNU, BBN, DMH, and DHPN, was performed as followers: DEN at a dose of 400 ppm was administered in a drinking water for 3 days from the very beginning of the experiment. We decided to perform DEN treatment in drinking water, as in the preliminary experiment too strong toxic effect was observed with *Ogg1*^−/−^ mice after the DEN intraperitoneal (i.p.) injection. After finishing DEN administration, four i.p. injections of MNU (20 mg/kg b.w.) were done (2 times/week), following by six subcutaneous (s.c.) injections of DMH (10 mg/kg b.w.) during Weeks 3 and 4. BBN at a dose of 0.05% was administered in drinking water for 4 weeks starting immediately after finishing the DEN treatment, and 0.1% DHPN was applied for 2 weeks in drinking water during Weeks 4 and 5. Mice in the control groups were administered saline as injections (i.p. or s.c.) or the tap water for drinking. Animals were observed every day and euthanized in case of becoming moribund during the study, or at the end of the experiment at Week 34. All surviving mice were killed under the isofluorene treatment and the DMBDD target organs, including liver, lung, kidneys, urinary bladder, small intestine and colon and thyroid gland, were immediately excised and fixed in 10% phosphate-buffered formalin, Thereafter, tissues were embedded in paraffin, sections of 4 μm in thickness were prepared and stained with hematoxylin and eosin (H & E) for the routine histology. We assessed the incidences of hyperplasia (HPL), adenoma and adenocarcinomas in the lungs, putative preneoplastic foci (PPFs), tumors, bile duct proliferation and biliary cysts in the liver, incidences of adenoma and adenocarcinoma in the small intestine and colon. Intestines were excised and intraluminally injected and fixed with 10% phosphate-buffered formalin.

### 4.4. Blood Biochemical Analysis

Blood was collected via the abdominal aorta from 7–9 mice per group per sex at the end of the study period after overnight fasting. Automatic analyzer (Olympus AJ-5200, Tokyo, Japan) was employed for the blood biochemical analysis to detect total protein (T-protein, g/dL), albumin/globulin ratio (A/G ratio), albumin (g/dL), total bilirubin (T-bil, mg/dL), aspartate aminotransferase (AST, IU/L), alanine aminotransferase (ALT, IU/L), γ-glutamyl transpeptidase (γ-GTP, IU/L), alkaline phosphatase (ALP, IU/L), triglycerides (TG, mg/dL), total cholesterol (T-chol, mg/dL), blood urea nitrogen (BUN, mg/dL), creatinine (mg/dL), chloride (Cl), sodium (Na), potassium (K), calcium (Ca) and inorganic phosphorus (IP) (mEq/L).

### 4.5. Statistical Analysis 

The statistical analysis of the significance of differences between mean values was performed with the StatLight-2000(C) program (Yukms corp, Tokyo, Japan). The inter group differences detected for the incidences of histopathological findings were analyzed with the by χ^2^ test Fisher’s exact probability test (two-sided). Kaplan–Meier analysis was used to examine the changes in survival rates of *Ogg1* knockout and wild type mice. Homogeneity of variance between of *Ogg1*^−/−^ and *Ogg1*^+/+^ groups was detected by the F test. Student’s *t*-test (two-sided) was applied in the case the data were homogeneous; otherwise, Welch test was used. *p* Values less than 0.05 were considered significant.

## Figures and Tables

**Table 1:** Final survival ratios, final body and relative organ weights of *Ogg1*^−/−^ and *Ogg1*^+/+^ mice.

Final Body and Organ Weights	*Ogg1*^−/−^				*Ogg1*^+/+^			
Group	G1	G2	G3	G4	G5	G6	G7	G8
Gender	Male	Male	Female	Female	Male	Male	Female	Female
Treatment	DMBDD	Control	DMBDD	Control	DMBDD	Control	DMBDD	Control
Effective No. of mice	20	20	20	20	20	20	20	20
No. of surviving animals ^a^ (%)	17(85)	20(100)	17(85)	20(100)	16(80)	19(95)	19(95)	20(100)
Final body weight (g) ^e^	29.0 ± 3.3	30.6 ± 1.9 ^c^	24.7 ± 1.5	25.0 ± 2.6 ^a^	26.7 ± 2.7 ****	33.8 ± 2.8	23.7 ± 1.6 ***	26.9 ± 2.5
Organ weights								
Liver (g)	1.48 ± 0.21 ***	1.26 ± 0.13 ^d^	1.07 ± 0.11 **^,b^	0.95 ± 0.13 ^d^	1.45±0.18 **	1.64 ± 0.22	0.94 ±0.15 ****	1.13 ± 0.12
Liver (%)	5.14 ± 0.71 ****	4.11 ± 0.32 ^d^	4.36 ± 0.40 ***^,a^	3.83 ± 0.38 ^b^	5.35 ± 0.77	4.93 ± 0.68	4.02 ± 0.53	4.22 ± 0.31
Kidneys (g)	0.43 ± 0.05 *	0.39 ± 0.07 ^d^	0.30 ± 0.02	0.28 ± 0.04 ^b^	0.40 ± 0.05 ****	0.53 ± 0.11	0.28 ± 0.04 **	0.34 ± 0.06
Kidneys (%)	1.51 ± 0.22 **	1.28 ± 0.20 ^c^	1.21 ± 0.07 *	1.14 ± 0.11 ^b^	1.50 ± 0.22	1.60 ± 0.30	1.22 ± 0.15	1.27 ±0.17
Spleen (g)	0.14 ± 0.15 *	0.06 ± 0.01 ^d^	0.07 ± 0.02 **	0.05 ± 0.01 ^d^	0.11 ± 0.08	0.09 ± 0.02	0.08 ± 0.02	0.08 ± 0.02
Spleen (%)	0.47 ± 0.49 *	0.20 ± 0.04 ^b^	0.30 ± 0.08 ***	0.23 ± 0.03 ^c^	0.39 ± 0.28	0.26 ± 0.08	0.35 ± 0.08	0.28 ± 0.05
Lungs (g)	0.25 ± 0.06 ****	0.17 ± 0.02	0.24 ± 0.04 ****	0.15 ± 0.02	0.27 ± 0.05 ****	0.19 ± 0.03	0.23 ± 0.04 ****	0.15 ± 0.03
Lungs (%)	0.90 ± 0.28 ****	0.56 ± 0.07	0.97 ± 0.16 ****	0.61 ± 0.08 ^a^	1.00 ± 0.22 ****	0.56 ± 0.09	0.98 ± 0.18 ****	0.56 ± 0.08

Data are Mean ± SD for the surviving animals at the end of the study. Relative organ weights were calculated with the following equation: Absolute organ weight/final body weight × 100. * *p* < 0.05; ** *p* < 0.01; *** *p* < 0.001; **** *p* < 0.0001: significantly different vs. the respective control groups of the same genotype. ^a^ *p* < 0.05; ^b^ *p* < 0.01; ^c^ *p* < 0.001; ^d^ *p* < 0.0001 significantly different vs. the respective *Ogg1*^+/+^ DMBDD-treated or control groups. ^e^ Final body weights of all survived mice at the termination of the experiment.

**Table 2:** Neoplastic and preneoplastic proliferative lesions in male and female *Ogg1*^−/−^ and *Ogg1*^+/+^ mice.

Incidence in Males (No. Mice (%))	*Ogg1*^−/−^		*Ogg1*^+/+^	
Group	G1	G2	G5	G6
Gender	Male	Male	Male	Male
Treatment	DMBDD	Control	DMBDD	Control
Effective No. mice	20	20	20	20
No. tumor-bearing mice (%)	20(100) ****	1(5)	17(85) ****	0
No. tumors/mouse	5.9 ± 3.5 ****^,a^	0.1 ± 0.2	3.5 ± 2.8 ****	0
**Lung**				
Adenoma	20(100) ****^,a^	1(5)	14(70) ****	0
Adenocarcinoma	7(35) **	0	2(10)	0
Total tumors	20(100) ****^,a^	1(5)	15(75) ****	0
HPL	20(100) ****	1(5)	20(100) ****	0
**Liver**				
HCA	4(20) ^(i)^	0	1(5)	0
Hemangioma	2(10)	0	1(5)	0
Hemangiosarcoma	3(15)	0	0	0
Total tumors	5(25) *	0	1(5)	0
Basophilic PPFs	2(10)	0	3(15)	0
Eosinophilic PPFs	0	0	1(5)	0
Mixed type PPFs	0	0	1(5)	0
**Kidneys**				
Tubular cell HPL	5(25) ^(^^i)^	1(5)	2(10)	1(5)
**Urinary Bladder**				
Papilloma	1(5)	0	0	0
TCC	1(5)	0	2(10)	0
Total tumors	2(10)	0	2(10)	0
Simple HPL	5(25) *	0	5(25)	1(5)
PN HPL	4(20) ^(i)^	0	2(10)	0
**Colon**				
Adenoma	4(20) ^(^^i)^	0	2(10)	0
Adenocarcinoma	1(5)	0	0	0
Total tumors	5(25) *	0	2(10)	0
**Small Intestine**				
Adenoma	3(15)	0	0	0
AdCa	1(5)	0	1(5)	0
Total tumors	4(20) ^(^^i)^	0	1(5)	0
**Forestomach**				
Squamous cell HPL	14(70) ****^,a^	0	7(35) **	0
**Glandular Stomach**				
Adenoma	1(5)	0	0	0
Adenomatous cell HPL	1(5)	0	1(5)	0
**Lymphoma/Leukemia**	3(15)	0	1(5)	0
**Skin/Subcutis**				
Fibrosarcoma	3(15)	0	0	0
**Incidence in Females (No. Mice (%))**	***Ogg1*** **^−/−^**		***Ogg1*** **^+/+^**	
Group	G3	G4	G7	G8
Gender	Female	Female	Female	Female
Treatment	DMBDD	Control	DMBDD	Control
Effective No. mice	20	20	20	20
No. tumor-bearing mice (%)	20(100) ****^,b^	0	8(40) **	0
No. tumors/mouse	4.6 ± 2.1 ****^,b^	0	0.7 ± 0.9 **	0
**Lung**				
Adenoma	19(95) ****^,b^	0	12(60) ****	0
AdCa	2(10)	0	1(5)	0
Total tumors	20(100) ****^,b^	0	12(60) ****	0
HPL	20(100) ****	1(5)	17(85) ****	0
**Liver**				
HCA	4(20) ^(i)^	0	0	0
Hemangioma	1(5)	0	1(5)	0
Hemangiosarcoma	1(5)	0	0	0
Total tumors	5(25) *	0	1(5)	0
Basophilic PPFs	1(5)	0	2(10)	0
**Kidneys**				
Renal cell adenoma	0	0	1(5)	0
Tubular cell HPL	2(10)	0	2(10)	0
**Urinary Bladder**				
TCC	1(5)	0	0	0
Simple HPL	6(30)	3(15)	2(10)	2(10)
PN HPL	3(15)	0	0	0
**Colon**				
Adenoma	2(10)	0	4(20) ^(^^i)^	0
**Small Intestine**				
Adenoma	1(5)	0	2(10)	0
**Forestomach**				
Squamous cell HPL	11(55) ***	1(5)	8(40) **	0
**Glandular Stomach**				
AdCa	1(5)	0	0	0
Adenomatous cell HPL	1(5)	2(10)	0	0
**Thyroid**				
Follicular cell Adenoma	0	0	1(5)	0
**Lymphoma/Leukemia**	4(20) ^(^^i)^	0	0	0
**T Cell Lymphoma**	0	0	1(5)	0
**Adrenals**				
Cortical HPL	1(5)	0	0	0

* *p* < 0.05; ** *p* < 0.01; *** *p* < 0.001; **** *p* < 0.0001 and ^(i)^ *p* = 0.05 vs. respective control mice of the same genotype. ^a^ *p* < 0.05; ^b^ *p* < 0.01; ^d^ *p* < 0.0001 vs. wild type control or DMBDD-treated mice. HPL, hyperplasia; HCA, hepatocellular adenoma; AdCa, adenocarcinoma; PN, papillary or nodular; TCC, transitional cell carcinoma, PPFs, putative preneoplastic foci.

**Table 3:** Blood biochemistry data of DMBDD-treated and control *Ogg1* knockout and wild type mice.

Parameter	*Ogg1*^−/−^		*Ogg1*^+/+^		*Ogg1*^−/−^		*Ogg1*^+/+^	
Group	G1	G2	G3	G4	G5	G6	G7	G8
Gender	Male	Female	Male	Female	Male	Female	Male	Female
Treatment	DMBDD	Control	DMBDD	Control	DMBDD	Control	DMBDD	Control
Effective No. mice	7	9	9	9	8	9	9	9
AST (IU/L)	88.9 ± 39.7 ^(i)^	52.3 ± 3.2	78.7 ± 14.6 **	58.3 ± 5.9 ^a^	75.1 ± 11.2 **	57.3 ± 9.1	65.1 ± 12.1 *	51.9 ± 5.6
ALT (IU/L)	98.7 ± 82.9 *	26.9 ± 9.7	50.7 ± 15.4 **	27.2 ± 7.4	61.4 ± 22.8 **	32.8 ± 7.9	42.0 ± 23.0 *	22.1 ± 2.8
ALP (IU/L)	298.6 ± 105.5	245.0 ± 53.7	535.6 ± 138.3	452.4 ± 88.3	382.3 ± 93.7 **	224.0 ± 35.9	472.4 ± 82.2 *	375.0 ± 93.0
γ-GTP (IU/L)	1.0 ± 0.0	1.0 ± 0.0	1.3 ± 0.7	1.0 ± 0.0	1.1 ± 0.4	1.0 ± 0.0	1.0 ± 0.0	1.0 ± 0.0
T-protein (g/dl)	4.5 ± 0.9	5.2 ± 0.3	5.1 ± 0.3	5.2 ± 0.2	5.0 ± 0.4	5.3 ± 0.2	5.0 ± 0.2 *	5.3 ± 0.3
Albumin (g/dL)	2.0 ± 0.5 *	2.5 ± 0.1	2.4 ± 0.3	2.6 ± 0.2	2.1 ± 0.2 **	2.4 ± 0.1	2.4 ± 0.2	2.6 ± 0.1
A/G ratio	0.8 ± 0.1	0.9 ± 0.1	0.9 ± 0.1	1.0 ± 0.1	0.7 ± 0.1 **	0.9 ± 0.1	1.0 ± 0.1	1.0 ± 0.1
T-BiL (mg/dL)	0.2 ± 0.0	0.2 ± 0.0	0.2 ± 0.0	0.2 ± 0.0	0.2 ± 0.0	0.2 ± 0.0	0.2 ± 0.0	0.2 ± 0.0
Na (mEq/L)	154.1 ± 2.7 *	153.4 ± 1.2 ^(i)^	152.2 ± 1.6 *	150.7 ± 1.9	154.9 ± 1.7 *	152.8 ± 1.2	153.0 ± 1.7 **	150.8 ± 1.4
K (mEq/L)	6.7 ± 1.3	6.1 ± 0.9	5.7 ± 0.9	6.2 ± 1.7	6.9 ± 0.7	6.6 ± 1.5	5.5 ± 0.9	5.3 ± 0.8
Cl (mEq/L)	109.7 ± 3.0	109.8 ± 1.8	111.0 ± 1.9	109.2 ± 2.3	111.3 ± 2.0 *	109.1 ± 1.5	112.7 ± 2.3 *	110.6 ± 2.2
Ca (mEq/L)	8.8 ± 0.5 ^a^	8.9 ± 0.5	8.6 ± 0.6	8.7 ± 0.5	9.4 ± 0.6	9.1 ± 0.4	8.7 ± 0.4	8.7 ± 0.3
IP (mEq/L)	10.3 ± 1.1	9.8 ± 1.1	9.5 ± 1.8 ^a^	9.4 ± 2.0 ^a^	9.5 ± 0.8	9.8 ± 1.0	7.8 ± 1.4	7.4 ± 0.9
T-Cholesterol (mg/dL)	78.3 ± 21.4	87.8 ± 9.1	65.3 ± 6.5	74.1 ± 17.3	78.0 ± 20.5 *	103.0 ± 29.8	59.4 ± 14.6 **	100.3 ± 9.6
TG (mg/dL)	114.0 ± 211.2	80.4 ± 32.1	33.4 ± 24.7	32.3 ± 16.1	72.9 ± 25.4	68.2 ± 41.3	27.1 ± 14.9	33.8 ± 16.6
BUN (mg/dL)	35.6 ± 6.0	33.4 ± 5.4	35.1 ± 4.0	32.1 ± 4.3	35.3 ± 3.5	31.4 ± 4.3	31.6 ± 7.2	26.3 ± 8.5
Creatinine (mg/dL)	0.05 ± 0.02 *	0.03 ± 0.02	0.05 ± 0.03 *	0.03 ± 0.02 ^b^	0.05 ± 0.03	0.04 ± 0.01	0.07 ± 0.02	0.07 ± 0.03

Values are means ± SD; * *p* < 0.05; ** *p* < 0.01; ^(^^i)^ *p* = 0.05 vs. respective control group of mice of same genotype. ^a^ *p* < 0.05; ^b^ *p* < 0.01 vs. respective wild type control or DMBDD group. TG, triglycerides; T-Bil, T-bilirubin; IP, inorganic phosphorus.

## Supplementary Materials

Supplementary materials can be found at www.mdpi.com/1422-0067/18/8/1801/s1.
