# Supplementary material for: Enhanced Susceptibility of Ogg1 Mutant Mice to Multiorgan Carcinogenesis
Source: Int J Mol Sci. 2017 Aug 18;18(8):1801. doi: 10.3390/ijms18081801 (PMC5578188; doi:10.3390/ijms18081801)
Supplement: Supplementary file 1 [file ijms-18-01801-s001.pdf]

Supplementary Materials: Enhanced Susceptibility of Ogg1 Mutant Mice to Multiorgan Carcinogenesis

Anna Kakehashi *, Naomi Ishii, Takahiro Okuno, Masaki Fujioka, Min Giand Hideki Wanibuchi

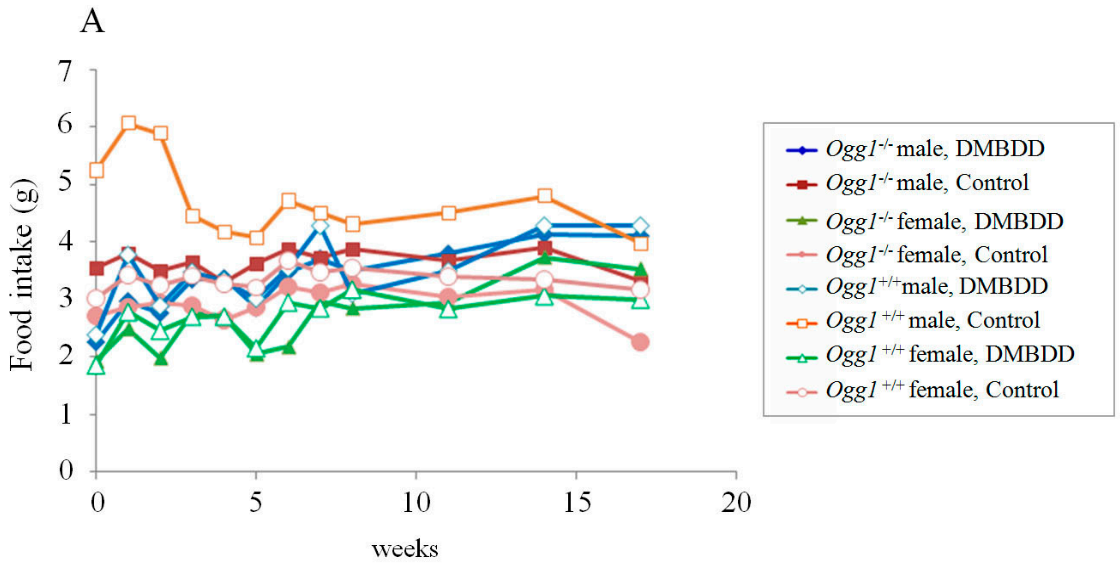

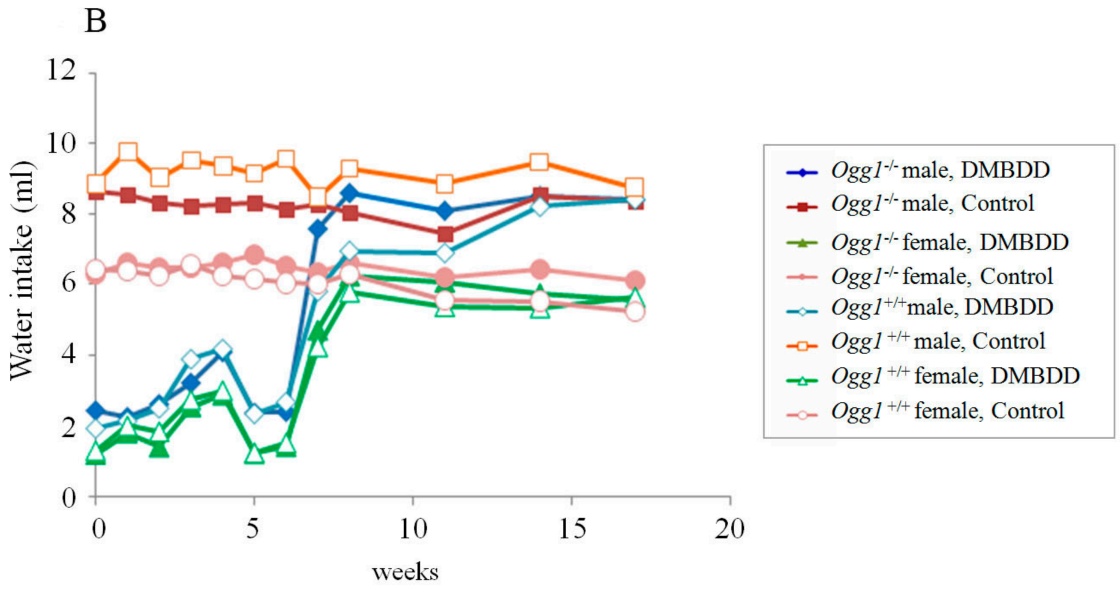

Figure S1. Food (**A**); and water (**B**) intakes of DMBDD-treated and control *Ogg1*$^{-/-}$ and *Ogg1*$^{+/+}$ mice.
